# Case report: Successful treatment with biologics in a pediatric patient with a severe inflammatory skin disease and novel *CARD14* mutation

**DOI:** 10.3389/fmed.2024.1360248

**Published:** 2024-02-05

**Authors:** Michał Niedźwiedź, Joanna Narbutt, Aleksandra Siekierko, Małgorzata Skibińska, Bartłomiej Kwiek, Dorota Sobolewska-Sztychny, Magdalena Ciążyńska, Katarzyna Poznańska-Kurowska, Antoni Gostyński, Aleksandra Lesiak

**Affiliations:** ^1^Department of Dermatology, Pediatric Dermatology and Oncology, Medical University of Lodz, Lodz, Poland; ^2^International Doctoral School, Medical University of Lodz, Lodz, Poland; ^3^Dermatology and Pediatric Dermatology Ward, Bieganski Hospital, Lodz, Poland; ^4^Medical Faculty, Lazarski University, Warsaw, Poland; ^5^Laboratory of Autoinflammatory, Genetic and Rare Skin Disorders, Medical University of Lodz, Lodz, Poland; ^6^Department of Dermatology, Maastricht University Medical Centre, Maastricht, Netherlands; ^7^GROW School for Oncology and Developmental Biology, Maastricht University, Maastricht, Netherlands

**Keywords:** *CARD14*, CAPE, biologics, adalimumab, ustekinumab, case report, *CARD14*: c.394A > T/− (Ile123Phe), *CARD14*-associated papulosquamous eruption

## Abstract

*CARD14* (caspase activation and recruitment domain) mutations have been associated with psoriasis vulgaris, psoriatic arthritis, generalized and palmoplantar pustular psoriasis, pityriasis rubra pilaris, and atopic dermatitis. We present a pediatric patient with a novel *CARD14*: c.394A > T/− (Ile123Phe) mutation, diagnosed with *CARD14*-associated papulosquamous eruption (CAPE), who was successfully treated with biological treatment.

## 1 Introduction

*CARD14* (caspase activation and recruitment domain) gene activates a group of interacting proteins known as nuclear factor-kappa-B (NF-κB), which regulate the activity of multiple genes, including those that control the immune responses and inflammatory reactions of the body (1, 2). Until now, *CARD14* mutations have been associated with psoriasis vulgaris (PsV), psoriatic arthritis (PsA), generalized and palmoplantar pustular psoriasis (GPP and PPP), pityriasis rubra pilaris (PRP), and atopic dermatitis (AD) (3–8).

In 2018, a new dermatological condition, *CARD14*-associated papulosquamous eruption (CAPE) was described for a group of patients with clinical features of psoriasis and PRP that also bear some resemblance to atopic dermatitis or even ichthyosis (6). Due to the limited data, there are no treatment guidelines for CAPE.

## 2 Case report

**Figure 1:**
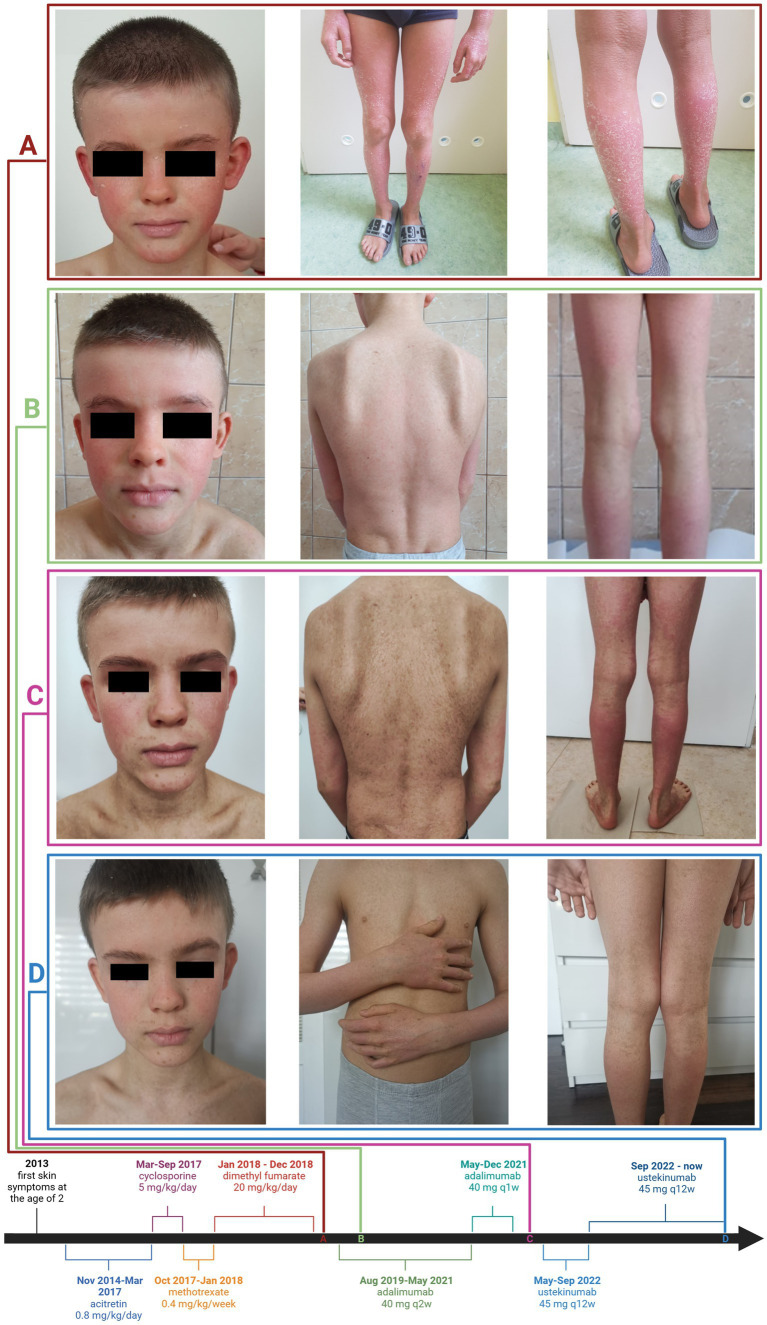
Skin symptoms of CAPE and case timeline. Photographs of the patient before treatment with adalimumab: well-demarcated pink-red patches and thin plaques, bilateral cheeks and chin with sparing of the infralabial area, involvement of the trunk, and extremities—erythema and significant scaling **(A)**, after 2 months on adalimumab therapy **(B)**, before ustekinumab therapy **(C)**, and after 13 months on ustekinumab therapy **(D)**.

We present an 11-year-old patient who developed skin problems by the age of 2. According to the patient’s parents, there was no significant history of skin diseases in the family. His diagnoses included psoriasis vulgaris and pityriasis rubra pilaris; however, no definitive diagnosis was made. The patient presented with well-demarcated pink-red patches and thin plaques involving bilateral cheeks and chin with sparing of the infralabial area and substantial involvement of the trunk and extremities in the form of erythema and significant scaling (Figure 1A). Histopathological examinations of several skin biopsies revealed features of PsV (parakeratosis mounded with neutrophils, hypogranulosis, and regular acanthosis) and PRP (alternating parakeratosis and orthokeratosis in a vertical and horizontal pattern, irregular acanthosis, and follicular plugging). He was treated with topical medications, including 0.5% betamethasone cream, 1% hydrocortisone cream, 0.1% mometasone furoate cream, systemic acitretin 0.8 mg/kg/day from 3 to 6 years old, cyclosporine 5 mg/kg/day for 4 months, methotrexate 0.4 mg/kg/week, and dimethyl fumarate 20 mg/kg/day for 11 months, all with poor response. Next-generation sequencing (NGS) panel targeted for mutations associated with ichthyosis, psoriasis, PRP, and EB revealed novel *CARD14*: c.394A > T/− (Ile123Phe) mutation. The gene variant has not been reported in the Human Gene Mutation Database, ClinVar, GnomAD, and ExAc databases. Bioinformatics analysis using the Alamut program software indicated that nucleotide A at position 394 and amino acid Ile at position 132 are highly evolutionarily conserved. The PolyPhen-2 algorithm and SIFT software analysis indicated the potentially pathogenic nature of the mutation. The patient was eventually diagnosed with *CARD14*-associated papulosquamous eruption (CAPE). Before initiating treatment with biologics. Children’s Dermatology Life Quality Index (CDLQI) and Family Dermatology Life Quality Index (FDLQI) questionnaires were filled out by the patient and his parents and assessed. Investigator Global Assessment (IGA) was also evaluated (see Table 1). Initial scores for CDLQI, FDLQI, and IGA were 17 (very large impact of the disease on the patient’s life), 28 (extremely large impact of the disease on the patient’s family life), and 4 (severe skin symptoms), respectively. He started biological therapy with tumor necrosis factor-α (TNF-α) inhibitor, adalimumab, with a dose of 40 mg SC every 14 days showing clinical improvement of his skin lesions for a period of 18 months without a total remission (Figure 1B). During the next 3 months, deterioration was observed and the frequency of administering the drug was modified to every 7 days (Figure 1C). Due to a lack of improvement, it was decided to change the biological treatment for ustekinumab, which is a monoclonal IgG1_k_ antibody that targets both IL-12 and IL-23 cytokines by binding to their shared p40 subunit. Initially, he was treated with a dose of 45 mg (1.14 mg per kg), then in the fourth week and then every 12 weeks thereafter, which is standard dosing for PsV and PsA (9). The patient additionally applied topical steroids, tacrolimus, and cholesterol ointment. Two months after the therapy initiation, the patient presented lower therapy effectiveness, and deterioration of skin lesions was observed. A possible cause could be the psychological trauma after a car accident that the patient was involved in. He suffered no physical injuries, and the treatment was uninterrupted. Therefore, based on available data in the literature and previously reported cases of patients with CAPE (Table 2), it was decided to increase the frequency of ustekinumab injections to every other 8 weeks with significant clinical improvement (Figure 1D). The patient did not report any side effects while undergoing therapy, and no side effects were observed by physicians. The patient’s parents also reported substantial improvement in his schoolwork and contact with peers, which is also noticeable in the FDLQI (3 points—small effect on the family’s life quality) and CDLQI (0 points—no effect on patient’s life quality) scores (Table 1).

**Table 1:** Presentation of the therapeutic course of the presented patient, taking into account the IGA, FDLQI, CDLQI scales, height, weight, biological agent, dose and frequency of the drug istration.

Date of assessment	IGA^1^	FDLQI^2^	CDLQI^3^	Height (cm)	Height percentile (%)	Weight (kg)	Weight percentile (%)	Biologics	Dose (mg)	Dose per body mass (dose mg/body mass kg)	Frequency (days)
**August 12, 2019**	**4**	**28**	**17**	138	89.2	27	56.6	QUALIFICATION			
**September 02, 2019**	**4**			139	98.6	28	63.8	Adalimumab	40	**1.43**	14
**October 7, 2019**	**3**			140	97.1	30	75	Adalimumab	40	**1.33**	14
**December 16, 2019**	**3**	**13**	**0**	141	94.5	30	71.1	Adalimumab	40	**1.33**	14
**March 30, 2020**	**2**			142	93.1	33	81	Adalimumab	40	**1.21**	14
**May 05, 2020**	**2**			143	93.9	34	83.1	Adalimumab	40	**1.18**	14
**July 13, 2020**	**2**			144	93.6	33	76	Adalimumab	40	**1.21**	14
**August 18, 2020**	**2**			145	94.4	35	82.4	Adalimumab	40	**1.14**	14
**October 14, 2020**	**2**			145	92.7	35	80	Adalimumab	40	**1.14**	14
**January 20, 2021**	**3**			146	91.4	34	70.6	Adalimumab	40	**1.18**	14
**February 16, 2021**	**4**			147	92.7	32	56.8	Adalimumab	40	**1.25**	14
**May 11, 2021**	**4**			149	94.1	33	57.8	Adalimumab	40	**1.21**	14
**August 03, 2021**	**3**			150	93.6	34	58.3	Adalimumab	40	**1.18**	7
**September 13, 2021**	**4**			ND	ND	ND	ND	Adalimumab	40	ND	7
**December 7, 2021**	**4**	**24**	**14**	ND	ND	ND	ND	Adalimumab	40	ND	7
**March 02, 2022**	**4**	**19**	**15**	ND	ND	ND	ND	QUALIFICATION			
**May 24, 2022**	**3**			157	96.9	39.5	62.8	Ustekinumab	45	**1.14**	28
**June 21, 2022**	**2**			158	97.4	38	59.6	Ustekinumab	45	**1.18**	84
**September 22, 2022**	**4**			159	96.9	39.5	61	Ustekinumab	45	**1.14**	84
**December 13, 2022**	**2**			160	96.5	41	62.7	Ustekinumab	45	**1.10**	56
**February 14, 2023**	**1**			162	97.3	42	63.3	Ustekinumab	45	**1.07**	56
**April 11, 2023**	**1**	**4**	**2**	163	97.3	43	64.1	Ustekinumab	45	**1.05**	56
**June 09, 2023**	**1**			164	97.2	43	64.5	Ustekinumab	45	**1.03**	56
**August 21, 2023**	**1**	**3**	**0**	165	96.7	44	60.3	Ustekinumab	45	**102**	56

^1^Investigator’s Global Assessment (IGA) interpretation: 0 = clear skin, 1 = almost clear skin, 2 = mild severity of lesions, 3 = moderate severity of lesions, 4 = severe skin lesions. ^2^Family and ^3^Children’s Dermatology Life Quality scores: 0–1 = no effect on life; 2–6 = small effect; 7–12 = moderate effect; 13–18 = very large effect; 19–30 = extremely large effect. The bold values and color shading inside is intended to facilitate the reception of the table.

**Table 2:** Summary of the patients diagnosed with CAPE and treated with ustekinumab.

Patients with CAPE treated with ustekinumab												
Publication	Described mutation	Age of onset	Facial involvement	Trunk involvement	Palmoplantar keratoderma	Follicular papules	Island of sparing	Family history for PsV, PsA, m/pGF, CAPE	Conventional treatment	Outcome	Biologic treatment	Outcome
Craiglow et al. (6)	c.349G > A, p.G117S (homozygous)	8 months	Yes	Yes	Yes	Yes	No	Positive	Isotretinoin	Partial	Ustekinumab 0.7 mg/kg q12w + methotrexate	Near complete
	c.34915G > C	2 years	Yes	Yes	Yes	No	No	Positive	Isotretinoin	Partial	Ustekinumab 1.1 mg/kg q12w	Near complete
	c.412G > A, p.E138K (*de novo*)	3 weeks	Yes	Yes	Yes	No	No	Negative	Acitretin	Minimal	Ustekinumab 0.87 mg/kg q12w	Partial
	c.467 T > C, p.L156P	6 months	Yes	Yes	Yes	No	Yes	Positive			Ustekinumab 1.2 mg/kg q8w	Near complete
	c.349G > A, p.G117S	1 year	Yes	Yes	No	No	No	Positive	Methotrexate	Partial	Ustekinumab 0.87 mg/kg q12w	Near complete
									Isotretinoin	Partial		
	c.371 T > C, p.L124P (*de novo*)	3 months	Yes	Yes	Yes	Yes	Yes	Negative	Methotrexate	Minimal	Ustekinumab 0.9 mg/kg q12w	Near complete
									Acitretin	Partial		
									Cyclosporine	Partial		
									Psoralen ultraviolet A	Worsening		
Signa et al. (21)	c.446 T > G, p.L149R (dizygotic twins)	9 months	Yes	Yes	No data	No data	No	Positive	Cyclosporine	Partial	Ustekinumab (2 mg/kg) q12w	Total remission
Nieto-Benito et al. (18)	c.277A > C, p.Lys(93Glu)	2 months	Yes	Yes	Yes	No data	Yes	Positive	Oral retinoids	No response	Ustekinumab 90 mg q12w	Near complete
Frare et al. (17)	c.1604A > G, p.Gln535Arg	10 months	Yes	Yes	No	No	Yes	Positive	Topical steroids	Minimal	Ustekinumab 45 mg q12w + methotrexate	Partial
	c.365 T > C, p.Met119Thr	3 months	Yes	Yes	Yes	Yes	Yes	Positive	Isotretinoin + mometasone	Partial		
									Methotrexate	Worsening	Ustekinumab 45 mg q12w	Worsening
											Ustekinumab 45 mg q8w	Near complete
Kiszewski et al. (25)	c.349 + 2 T > C	5 months	Yes	Yes	Yes	Yes	Yes	No data	Cyclosporine	No response	Ustekinumab 10.8 mg q2w	Near complete
									Methotrexate	Minimal		
Noguiera et al. (24)	c.349 + 5G > C	8 months	Yes	Yes	No data	Yes	Yes	Positive	Topical agents	Minimal	Ustekinumab 0.75–1.0 mg q8w	Near complete
Niedźwiedź et al. (26)	c.394A > T/− (Ile123Phe)	2 years of age	Yes	Yes	Yes	Yes	Yes	Negative	Systemic acitretin, cyclosporine, methotrexate, dimethyl fumarate	No or poor response	Adalimumab 40 mg q1-2w	Partial with decreased response to the drug
											Ustekinumab 1.0–1.18 mg/kg q8-12w	Near complete

The color shading is intended to facilitate the reception of the table.

## 3 Discussion

The *CARD14* gene provides instructions for making a protein that activates a group of interacting proteins known as nuclear factor-kappa-B (NF-κB). The NF-κB protein complex is responsible for the activation and regulation of multiple genes, including those that are responsible for inflammatory reactions. The NF-κB protein complex also protects cells from certain signals that would otherwise cause them to undergo apoptosis (1, 10, 11). NF-κB signaling plays a vital role in regulating inflammatory reactions in the skin and in promoting the *survival* of the skin (1, 10–12). *CARD14* gain-of-function (GOF) mutations are linked with clinical features of PsV and PRP, while loss-of-function mutations are associated with atopic dermatitis. GOF mutation in *CARD14* results in heightened nuclear factor κB (NF-κB) signaling (6, 11, 12). Elevated NF-κB activity leads to increased levels of chemokines such as IL-8 and CCL20, which, in turn, lead to the recruitment and differentiation of inflammatory cells, including the production of IL-23 by dendritic cells and IL-17 and IL-22 by T cells.

**Figure 2:**
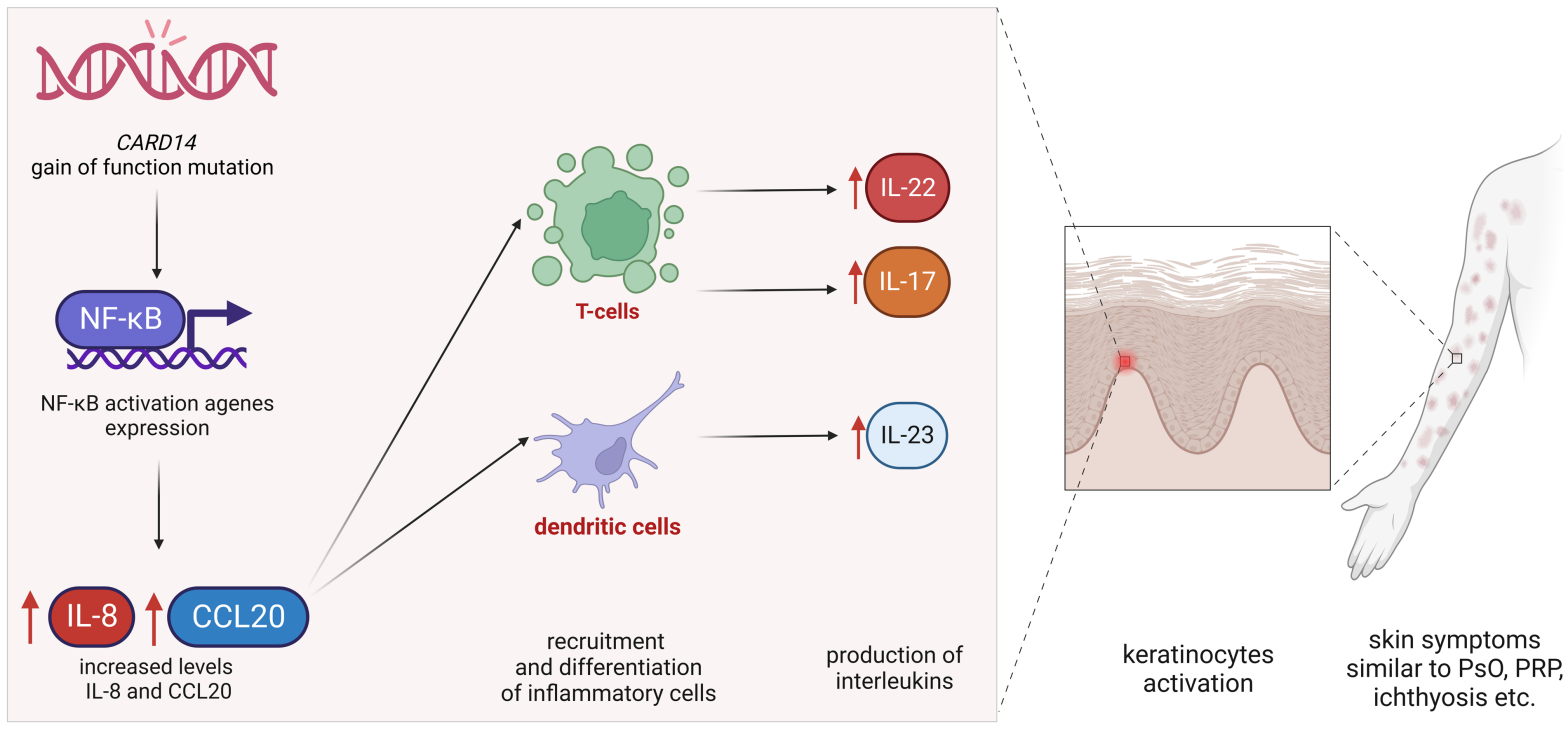
Simplified pathomechanism of *CARD14* gain-of-function mutations. GOF mutation in *CARD14* results in heightened nuclear factor κB signaling. Elevated NF-κB activity leads to increased levels of chemokines, such as IL-8 and CCL20, which, in turn, lead to the recruitment and differentiation of inflammatory cells, including the production of IL-23 by dendritic cells and IL-17 and IL-22 by T cells. Increased levels of these interleukins cause parakeratosis leading to skin symptoms similar to psoriasis, pityriasis rubra pilaris, and/or ichthyosis.

The role of *CARD14* in the pathogenesis of several inflammatory skin conditions was initially described through publications of familial cases of PsV and PRP (13–15). Gál et al. (16) identified several *CARD14* variants in almost half of their cases of PRP, but no correlation was found between the therapeutic response and the genetic background, which could have been due to a limited number of patients. To date, there are several reports that indicate that various *CARD14* mutations may lead to autoinflammatory skin diseases such as plaque, pustular and/or erythrodermic types of psoriasis, pityriasis rubra pilaris, ichthyosis, and psoriatic arthritis (Figure 2). Dominant loss of function mutations in *CARD14* resulted in an unusually severe form of atopic dermatitis (11).

In 2018, a new term, CARD-14-associated papulosquamous eruption (CAPE) was introduced by Craiglow et al. (6) to describe a group of patients with clinical features of psoriasis and pityriasis rubra pilaris (PRP) bearing some resemblance to atopic dermatitis or even ichthyosis. There was no definite diagnosis in those patients, and topical or systemic treatment, including cyclosporin or methotrexate, was unsuccessful. All patients with CAPE had *CARD14* mutations. Clinical characteristics of patients with CAPE are as follows: (1) young onset of skin symptoms, (2) facial involvement with well-demarcated pink-red plaques involving the cheeks, chin, and ears with sparing of the infralabial region, (3) palmoplantar keratoderma, (4) trunk involvement, (5) follicular papules, and (6) island of sparing (6, 17). Patients diagnosed with CAPE are reported to present a low quality of life and tend to present psychological symptoms such as depression (18).

Histopathological examinations are reported to be not diagnostic enough because biopsies showed conflicting microscopic pictures, which also occurred in our patient (19). Ring et al. evaluated biopsies of skin lesions from patients diagnosed with CAPE and compared them with biopsies of PsV and PRP patients (18). In the studied skin samples, CAPE shared more histopathologic features with PRP than with psoriasis, including checkerboard parakeratosis and orthokeratosis, acanthosis, follicular plugging, and similar thickness of the epidermis below the stratum corneum and a lack of relative suprapapillary plate thinning. CAPE samples demonstrated regular psoriasiform acanthosis with elongated rete ridges in contrast to PRP specimens. Similar to PsV, CAPE also lacked acantholysis, while approximately half of the PRP specimens presented with acantholysis.

Patients with CAPE are reported to present poor responses to conventional topical and systemic therapy such as acitretin, cyclosporine, or methotrexate (6, 17). The overlapping stimulation of the IL-23/Th17 axis caused by *CARD14* mutations indicates that blocking this pathway may be the best treatment option for patients with CAPE. Biologics, such as ustekinumab, guselkumab, secukinumab, and ixekizumab, are reported to present beneficial treatment responses in *CARD14*-related diseases (6, 20, 21).

Based on the known effects of GOF mutations in the *CARD14* gene and its effects on NF-κB, ustekinumab appears to be a pathogenesis-based treatment for CAPE, as shown by the several clinical responses in published case reports (Table 2). In available literature data including our patient, 11 of 12 described patients treated with high doses of ustekinumab responded to biological treatment with at least a good response. Patients with CAPE may require a more frequent or higher dose of biologics to achieve remission than patients with psoriasis. Nieto-Benito et al. (18) describe a 36-year-old man with CAPE who was treated for 14 years for ichthyosis and progressive symmetric erythrokeratoderma with acitretin with poor response. After a genetic investigation, it was decided to start therapy with ustekinumab with a good response.

Despite promising data, long-term follow-up of patients treated with these biological molecules is still lacking. Our presented patient with CAPE is currently undergoing treatment with high doses of ustekinumab for 21 months and is showing clinical improvement; however, the dosing and the frequency of medicine administration should be assessed individually (22–24). The remaining question is whether *CARD14* mutations can be associated with severe inflammatory skin condition resistance to treatment.

A limitation of our study is a lack of histopathological images of performed biopsies. These were evaluated by a non-university, external company and provided only the descriptions of the images. We also did not perform molecular assessment during the course of the treatment for levels of inflammatory markers, such as IL-17, IL-22 and IL-23, or TNF-α.

## 4 Conclusion

Patients with CAPE share clinical findings, mostly similar to psoriasis and/or pityriasis rubra pilaris. Patients with gain-of-function *CARD14* mutations and diagnosed with CARD-14-associated papulosquamous eruption present similar phenotypes such as young onset of the skin symptoms, facial involvement with well-demarcated pink-red plaques involving the cheeks, chin, and ears with sparing of the infralabial region, palmoplantar keratoderma, trunk involvement, follicular papules, and the island of sparing. Patients with severe inflammatory skin conditions and presenting phenotypical features, who do not respond to standard treatment, should be considered for genetic investigations for *CARD14* mutations. Patients diagnosed with CAPE had poor responses to conventional psoriasis treatment, acitretin, cyclosporine, or methotrexate. Unfortunately, there are still not enough data to establish generally accepted therapeutic guidelines for *CARD14*-related dermatological conditions; however, treatment with high doses of biologics targeting psoriasis pathways, IL-23 and IL-17, such as ustekinumab, shows promising results.

## Data availability statement

The original contributions presented in the study are included in the article/supplementary material, further inquiries can be directed to the corresponding author.

## Ethics statement

Written informed consent was obtained from the minor’s legal guardian/next of kin for the publication of any potentially identifiable images or data included in this article.

## Author contributions

MN: Data curation, Formal analysis, Visualization, Writing – original draft, Writing – review & editing, Conceptualization. JN: Funding acquisition, Supervision, Writing – review & editing. AS: Data curation, Writing – original draft. MS: Supervision, Writing – review & editing. BK: Writing – review & editing, Data curation. DS-S: Data curation, Writing – original draft. MC: Supervision, Writing – review & editing. KP-K: Data curation, Writing – review & editing. AG: Supervision, Writing – review & editing. AL: Formal analysis, Funding acquisition, Supervision, Writing – review & editing.
